# An extracellular domain of the EsaA membrane component of the type VIIb secretion system: expression, purification and crystallization

**DOI:** 10.1107/S2053230X1901495X

**Published:** 2019-11-20

**Authors:** Nicole Mietrach, Andreas Schlosser, Sebastian Geibel

**Affiliations:** a Institute for Molecular Infection Biology, Julius-Maximilians-University Würzburg, Josef Schneider Strasse 2, 97080 Würzburg, Germany; b Rudolf Virchow Center for Experimental Biomedicine, Julius-Maximilians-University Würzburg, Josef Schneider Strasse 2, 97080 Würzburg, Germany

**Keywords:** ESAT-6-like secretion system, ESS, type VII secretion system, EsaA, extracellular domain, *Staphylococcus aureus* USA300

## Abstract

The membrane protein EsaA is a conserved component of the type VIIb secretion system. Limited proteolysis of purified EsaA from *Staphylococcus aureus* USA300 identified a stable 48 kDa fragment, which was mapped by fingerprint mass spectrometry to an uncharacterized extracellular segment of EsaA. Analysis by circular dichroism spectroscopy showed that this fragment folds into a single stable domain made of mostly α-helices with a melting point of 34.5°C. Size-exclusion chromatography combined with multi-angle light scattering indicated the formation of a dimer of the purified extracellular domain. Octahedral crystals were grown in 0.2 *M* ammonium citrate tribasic pH 7.0, 16% PEG 3350 using the hanging-drop vapor-diffusion method. Diffraction data were analyzed to 4.0 Å resolution, showing that the crystals belonged to the enantiomorphic tetragonal space groups *P*4_1_2_1_2 or *P*4_3_2_1_2, with unit-cell parameters *a* = 197.5, *b* = 197.5, *c* = 368.3 Å, α = β = γ = 90°.

## Abstract 2

The biophysical characterization and crystallization of an extracellular domain of the EsaA membrane component of the type VIIb secretion system is described.

## 1. Introduction   

Type VII secretion systems are used by a broad range of Gram-positive bacteria to secrete effector proteins across their cell walls. While type VIIa secretion systems (also termed ESX secretion systems) are found in Actinomycetes and have been linked to tuberculosis, type VIIb systems (also termed ESS secretion systems) are found in Firmicutes and have been implicated in *Staphylococcus aureus* infections as well as in bacterial competition (Gröschel *et al.*, 2016 ▸). The two systems have two homologous components in common: (i) one or more effector proteins of the WXG100 protein family and (ii) an ATPase that recognizes the substrates in the cytoplasm and energizes their transport across the membrane (Unnikrishnan *et al.*, 2017 ▸). Additional system-specific proteins are required for function. The type VIIb secretion system encodes four conserved membrane components (EssA, EssB, EssC and EsaA), which are necessary and sufficient for the secretion of effector proteins (Burts *et al.*, 2005 ▸; Kneuper *et al.*, 2014 ▸). Recent studies have indicated that these components form a complex embedded in the membrane, which was suggested to compose the secretion machine (Aly *et al.*, 2017 ▸; Mielich-Süss *et al.*, 2017 ▸). Biochemical studies have shown that two of these components (EssB and EsaA) assemble into a complex in the cell envelope (Ahmed *et al.*, 2018 ▸), where their assembly is mediated by flotillin, a constitutent of lipid rafts (Mielich-Süss *et al.*, 2017 ▸). The EsaA membrane component (115 kDa) is unique to type VIIb secretion systems; it is predicted to have six transmembrane α-helices and contains an uncharacterized extracellular segment, which was identified in a molecular-shaving experiment (Dreisbach *et al.*, 2010 ▸). Here, we report the expression, purification and crystallization of a stable 48 kDa domain covering most of the extracellular segment of EsaA.

## 2. Materials and methods   

### 2.1. Protein expression, purification and biophysical characterization   

#### 2.1.1. Cloning of EsaA, EsaAex_1 and EsaAex_2   

The pASK-IBA3C vector was linearized by PCR (primers X1/X2). After codon-optimization for *Escherichia coli*, the *esaA* gene and the fragment *esaAex_1* (coding for amino acids 47–804, which correspond to the predicted soluble part of EsaA) were cloned into the linearized pASK-IBA3C vector using Phusion polymerase (Invitrogen) and In-Fusion cloning (Clontech) (primer pairs X3/X4 and X5/X6), respectively. All primers are listed in Table 1 ▸. The DNA segment corresponding to the proteolytic fragment of *esaAex_1* (*esaAex_2*; amino acids 275–689) was amplified using primer pair X7/X8. In-Fusion cloning was used to ligate the amplified *esaAex_2* fragment into pET-16b vector including a Tobacco etch virus (TEV) cleavage site to produce the construct pET-16b-HIS-TEV-*esaAex_2* (primer pair X9/X10).

#### 2.1.2. Expression and purification of EsaA and EsaAex_1   

*E. coli* BL21 Star cells were transformed with either pASK-IBA3C-*esaA* or pASK-IBA3C-*esaAex_1* and were grown in LB medium supplemented with 25 µg ml^−1^ chloramphenicol. Protein expression was induced by the addition of anhydro­tetracycline (AHT; IBA Life Sciences) to a final concentration of 2 µg ml^−1^ at an optical density (OD_600_) of 0.6. Bacteria transformed with pASK-IBA3C-*esaA* were grown for 20 h at 18°C, whereas bacteria transformed with pASK-IBA3C-*esaAex_1* were grown for 20 h at 26°C. The bacteria were harvested by centrifugation (4000*g*, 4°C). The bacterial pellets were resuspended in 50 m*M* Tris–HCl pH 8.0, 300 m*M* NaCl, 3 m*M* DTT and lysed by three passages through an EmulsiFlex-C3 homogenizer (Avestin).

For the purification of EsaA, the bacterial membranes were isolated by ultracentrifugation (100 000*g*, 1 h, 4°C). The membrane fraction of EsaA was resuspended in 50 m*M* Tris–HCl pH 8.0, 300 m*M* NaCl, 3 m*M* dithiothreitol (DTT) and incubated in 0.5% *n*-dodecyl-β-d-maltopyranoside (DDM) for 1 h at 4°C. Insoluble material was removed by ultracentrifugation (100 000*g*, 1 h, 4°C) and the supernatant was loaded onto a Strep-Tactin column (1 ml; IBA Life Sciences) equilibrated with 50 m*M* Tris–HCl pH 8.0, 300 m*M* NaCl, 3 m*M* DTT, 0.05% DDM. The column was washed with equilibration buffer until the UV baseline was reached, followed by elution in the same buffer supplemented with 2.5 m*M* d-desthiobiotin. The peak fractions were collected and concentrated to a volume of 0.5 ml using 100 kDa centrifugal concentrators (Millipore). The sample was loaded onto a Superose 6 Increase column (GE Healthcare) equilibrated with 20 m*M* Tris–HCl pH 8.0, 150 m*M* NaCl, 3 m*M* DTT, 0.05% DDM.

For the purification of EsaAex_1, the bacteria were disrupted as described above and the lysate was clarified by ultracentrifugation (100 000*g*, 1 h, 4°C). The supernatant containing EsaAex_1 was loaded onto a Strep-Tactin column (1 ml; IBA Life Sciences) equilibrated with 50 m*M* Tris–HCl pH 8.0, 300 m*M* NaCl, 3 m*M* DTT. The column was washed with equilibration buffer until the UV baseline was reached, followed by elution in the same buffer supplemented with 2.5 m*M* d-desthiobiotin. The peak fractions were collected and concentrated to a volume of 2 ml using 10 kDa centrifugal concentrators (Millipore). The sample was loaded onto a Sepharose 300 column (GE Healthcare) equilibrated with 20 m*M* Tris–HCl pH 8.0, 150 m*M* NaCl, 3 m*M* DTT. The peak fractions were concentrated using a 10 kDa concentrator and used in proteolysis experiments.

#### 2.1.3. Expression and purification of EsaAex_2   

For the purification of EsaAex_2, *E. coli* BL21 Star cells harboring pET-16b-*esaAex_2* were grown in Luria–Bertani medium supplemented with 100 µg ml^−1^ ampicillin at 37°C. Protein expression was induced by the addition of isopropyl β-d-1-thiogalactopyranoside (IPTG) to a final concentration of 1 m*M* at an OD_600_ of 0.6. The bacteria were grown for 16 h at 26°C and harvested by centrifugation (4000*g*, 15 min). The bacterial pellet was resuspended in buffer *A* (50 m*M* Tris–HCl pH 8, 150 m*M* NaCl). The bacteria were disrupted as described above and the cell debris was removed by ultracentrifugation (100 000*g*, 1 h, 26°C). The supernatant was supplemented with 20 m*M* imidazole and loaded onto a HisTrap HP column (GE Healthcare) equilibrated with buffer *A*. A wash step was applied using 20% buffer *B* (50 m*M* Tris–HCl pH 8, 250 m*M* imidazole) until the UV absorbance reached the baseline before step elution with 100% buffer *B*. The His tag was then cleaved using TEV protease (1:10). The sample was dialyzed in buffer *A*, applied onto a 5 ml Ni–NTA column and the flowthrough was collected. EsaAex_2 was concentrated to a volume of 2 ml using a Millipore centrifugation device (10 kDa cutoff) and subjected to size-exclusion chromatography using a Sephacryl 300 column equilibrated with buffer *C* (150 m*M* NaCl, 20 m*M* Tris–HCl pH 8).

#### 2.1.4. Limited proteolysis of EsaAex_1   

The extracellular domain of EsaA (EsaAex_1; amino acids 47–804) was purified and subjected to limited proteolysis. 150 µg EsaAex_1 was incubated with 1.5 µg trypsin for 1 h at room temperature. Samples were taken every 15 min and the reaction was stopped with 3× protease-inhibitor cocktail (Roche). The samples were analyzed by SDS–PAGE. The protein band at 48 kDa was excised and sent for mass-spectrometric analysis.

#### 2.1.5. Analysis of the proteolysed EsaAex_1 by Nano LC-MS/MS   

After limited proteolysis, the proteolytic fragments were resolved by SDS–PAGE and Coomassie-stained and the EsaAex_1 band was excised. The excised gel band was destained with 30% acetonitrile in 0.1 *M* ammonium bicarbonate pH 8, shrunk with 100% acetonitrile and dried in a vacuum concentrator (Concentrator 5301, Eppendorf, Germany). Digests were performed with 0.1 µg elastase per gel band overnight at 37°C in 0.1 *M* ammonium bicarbonate pH 8. After removing the supernatant, the peptides were extracted from the gel slices with 5% formic acid and the extracted peptides were pooled with the supernatant.

Nano LC-MS/MS analyses were performed on an Orbitrap Fusion (Thermo Scientific) equipped with an EASY-Spray ion source and coupled to an EASY-nLC 1000 (Thermo Scientific). The peptides were loaded onto a trapping column (2 cm × 75 µm internal diameter, PepMap C18, 3 µm particles, 100 Å pore size) and separated on an EASY-Spray column (25 cm × 75 µm internal diameter, PepMap C18, 2 µm particles, 100 Å pore size) with a 30 min linear gradient from 3% to 30% acetonitrile and 0.1% formic acid.

Both MS and MS/MS scans were acquired in the Orbitrap analyzer with resolutions of 60 000 for MS scans and 15 000 for MS/MS scans. HCD fragmentation with 35% normalized collision energy was applied. A top speed data-dependent MS/MS method with a fixed cycle time of 3 s was used. Dynamic exclusion was applied with a repeat count of 1 and an exclusion duration of 30 s; singly charged precursors were excluded from selection. The minimum signal threshold for precursor selection was set to 50 000. Predictive automated gain control (AGC) was used with AGC target values of 2 × 10^5^ for MS scans and 5 × 10^4^ for MS/MS scans. EASY-IC was used for internal calibration.

Database searching was performed against a custom database containing the protein sequence of interest with the *PEAKS* 8.0 software (Bioinformatics Solutions) using the following parameters: parent mass tolerance, 8 p.p.m.; fragment mass tolerance, 0.02 Da; enzyme, none; variable modifications, oxidation (M), pyroglutamate (N-terminal Q), protein N-terminal acetylation. Results were filtered to a 1% peptide-to-spectrum match false-discovery rate by the target-decoy approach.

#### 2.1.6. Size-exclusion chromatography–multi-angle light scattering (SEC-MALS) of EsaAex_2   

SEC-MALS experiments were performed on a Superdex 200 10/300 GL column (GE Healthcare) coupled to a Dawn 8+ MALS detector and an Optilab T-rEX refractive-index detector (Wyatt Technology, Santa Barbara, California, USA). The column was equilibrated with 150 m*M* NaCl, 20 m*M* Tris–HCl pH 8.0, 3 m*M* DTT. EsaAex_2 was concentrated to 4 mg ml^−1^ and a sample volume of 100 µl was loaded onto the column. Size-exclusion chromatography was run at a flow rate of 0.5 ml min^−1^. The molecular mass of EsaAex_2 was determined using the *ASTRA* 6 software (Wyatt Technology).

#### 2.1.7. Circular dichroism (CD) and thermal unfolding experiments   

CD spectroscopy was performed using a Jasco J-810 spectropolarimeter. Spectra were recorded from 195 to 260 nm at a scanning speed of 50 nm min^−1^ with a response time of 2 s and a band width of 1 nm at 4°C. Thermal unfolding experiments of EsaAex_2 were conducted at 224 nm (bandwidth 2 nm, response time 16 s) starting at 4°C and heating to 70°C (at a heating rate of 1 K min^−1^). 5 µ*M* EsaAex_2 in 50 m*M* phosphate buffer pH 8.0 was used.

### 2.2. Crystallization   

After size-exclusion chromatography, the purest peak fractions of EsaAex_2 were pooled and concentrated to 5 mg ml^−1^ using centrifugal concentrators (Millipore, 10 kDa cutoff). 1.5 µl protein solution and 1.5 µl reservoir solution were mixed in a 1:1 ratio and placed on 22 mm circular siliconized cover slides (Jena Bioscience). Hanging-drop crystallization experiments were performed in Crystalgen plates using a reservoir volume of 600 µl. Crystals appeared after 4–5 days and grew to dimensions of ∼0.2 × 0.2 mm. Before flash-cooling, crystals were cooled to 4°C in their mother liquor for 24 h. Pre-cooled reservoir solution containing 25% glycerol was added gradually to the crystals until a final concentration of 25%(*v*/*v*) glycerol was reached in the crystallization drop, and the crystals were flash-cooled in liquid nitrogen. Crystallization information is summarized in Table 2 ▸.

### 2.3. Data collection and processing   

Crystals of EsaAex_2 were flash-cooled in liquid nitrogen and diffraction experiments were performed on beamline ID30-A3 at the European Synchrotron Radiation Facility (ESRF). The beam transmission was set to 25.2%. The collected data were processed using the *XDS* software package (Kabsch, 2010 ▸). *POINTLESS* from the *CCP*4 package (Winn *et al.*, 2011 ▸) was used for space-group determination (Evans, 2006 ▸). The Matthews coefficient (Matthews, 1968 ▸) and the solvent content were calculated using *MATTHEWS_COEF*. Attempts to solve the structure by molecular replacement failed.

## 3. Results and discussion   

**Figure 1:**
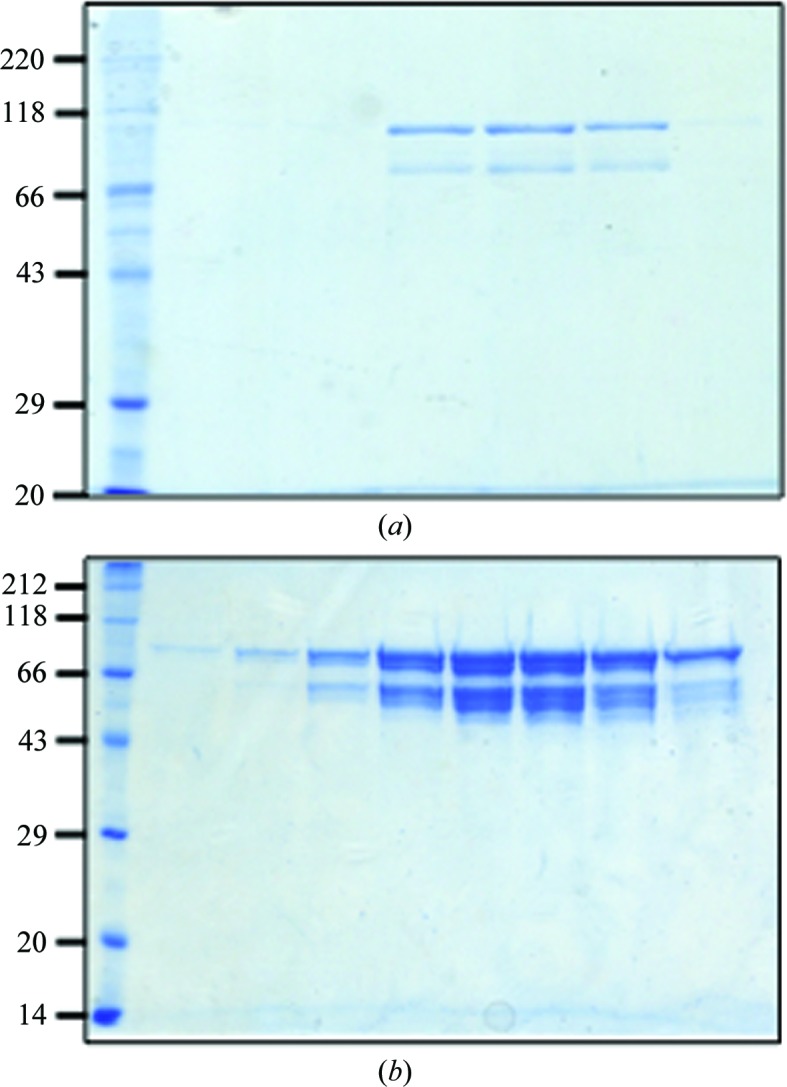
SDS–PAGE of (*a*) EsaA and (*b*) EsaAex_1 after size-exclusion chromatography. Molecular-mass markers are labeled in kDa.

**Figure 2:**
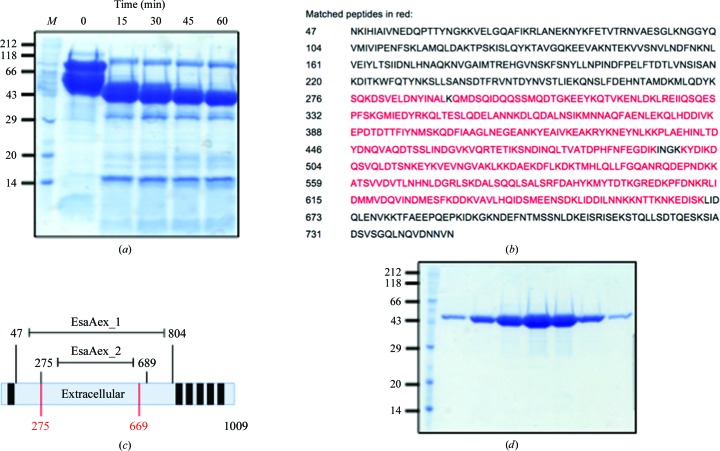
(*a*) SDS–PAGE analysis of EsaAex_1 after limited proteolysis. Lane *M* contains molecular-mass markers (labeled in kDa). (*b*) Sequence of EsaAex_1: the peptide coverage detected by fingerprint mass spectrometry (red) indicates the protein boundaries of the excised band at 43 kDa. (*c*) Schematic of the full-length EsaA protein showing the EsxAex_1 and EsaAex_2 boundaries and the MS fingerprint (red). (*d*) SDS–PAGE of EsaAex_2 after size-exclusion chromatography. Molecular-mass markers are labeled in kDa.

Purified full-length EsaA (amino acids 1–1009) and the variant EsaAex_1 (amino acids 47–804), which composed the predicted soluble part of the membrane protein, showed similar degradation patterns (Figs. 1 ▸ *a* and 1 ▸ *b*). In order to identify a stable protein core, EsaAex_1 was subjected to limited proteolysis (Fig. 2 ▸ *a*). After the addition of trypsin, samples were taken over the course of 1 h. SDS–PAGE analysis showed a stable proteolytic product of ∼48 kDa (Fig. 2 ▸ *a*). The boundaries of the protein fragment were determined by fingerprint mass spectrometry (amino acids 275–669; Fig. 2 ▸ *b*) and matched an extracellular segment of EsaA which was identified in molecular-shaving experiments of whole cells from different *S. aureus* strains (Dreisbach *et al.*, 2010 ▸). Since no crystals of this fragment (amino acids 275–669) could be obtained, a variant (EsaAex_2; amino acids 275–689) was cloned which preserved a predicted α-helix (597–683) at the C-terminus and was purified to homogeneity using nickel-affinity and size-exclusion chromatography (Figs. 2 ▸ *c* and 2 ▸ *d*).

**Figure 3:**
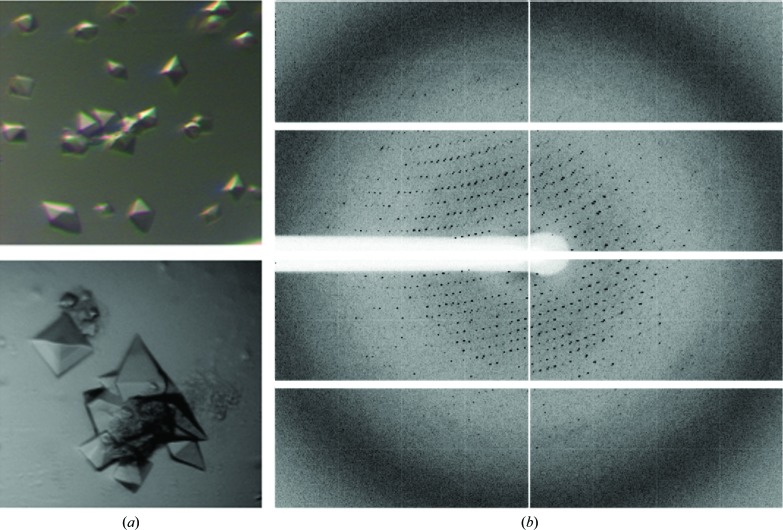
(*a*) Octahedral crystals and (*b*) diffraction image of EsaAex_2.

Octahedral crystals of EsaAex_2 were grown in hanging drops at 293 K (Fig. 3 ▸ *a*). However, the diffraction of these crystals varied strongly and was limited to ∼5 Å resolution. Cooling the crystals to 4°C 24 h before flash-cooling improved the diffraction of some, but not all, crystals to 4.0 Å resolution (Fig. 3 ▸ *b*; Table 3 ▸). The crystals belonged to the enantiomorphic tetragonal space group *P*4_1_2_1_2 or *P*4_3_2_1_2, with unit-cell parameters *a* = 197.5, *b* = 197.5, *c* = 368.3 Å, α = β = γ = 90°. Based on Matthews coefficient calculations (*V*_M_ = 2.34 Å^3^ Da^−1^; solvent content 47.55%), there are 16 molecules in the asymmetric unit (Matthews, 1968 ▸). Attempts to solve the structure by molecular replacement failed owing to a lack of homologous structures. Heavy metal atom screens are currently being carried out to obtain phases.

**Figure 4:**
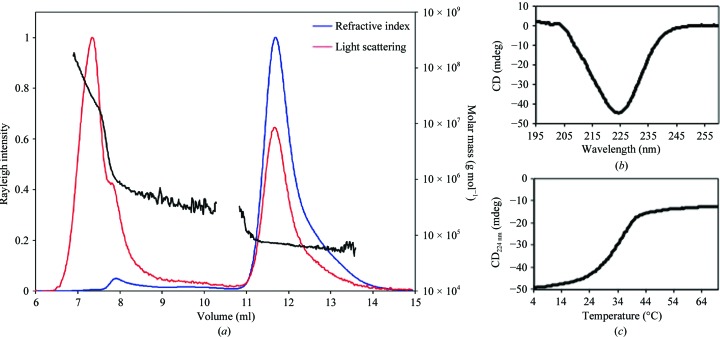
Biophysical characterization of EsaAex_2. (*a*) SEC-MALS analysis using a Superdex 200 10/300 GL column, (*b*) CD spectrum and (*c*) melting curve.

Size-exclusion chromatography combined with multi-angle light scattering indicated the formation of a dimer (Fig. 4 ▸ *a*). Circular dichroism spectroscopy revealed that EsaAex_2 contains 70% α-helices and 4% β-sheets (Perez-Iratxeta & Andrade-Navarro, 2008 ▸; Fig. 4 ▸ *b*). Melting-curve analysis showed a single transition, suggesting that the extracellular fragment is a single domain with a melting temperature of 34.5°C (Fig. 4 ▸ *c*).

## Figures and Tables

**Table 1:** Macromolecule-production information

Source organism	*S. aureus* USA300
DNA source	Synthesized DNA, codon-optimized for *E. coli*
X1	TGGAGCCACCCGCAGT
X2	TTTTTGCCCTCGTTATCTAGATTTTTGTCGA
X3	TAACGAGGGCAAAAAATGAAAAAGAAAAATTGGATTTAC
X4	GTGGCTCCAAGCGCTAATCAGGCGTTCCTTTTTGA
X5	TAACGAGGGCAAAAAATGAACAAAATCCATATCGCA
X6	GTGGCTCCAAGCGCTTCCACCAACCGGGTTCGACATAAAG
X7	TACTTCCAATCCGGAATGTCACAAAAAGACTCGGT
X8	TAGTTATTGCTCAGCTTATTTCGGTTCTTGCGGTT
X9	GCTGAGCAATAACTAGCATAAC
X10	TCCGGATTGGAAGTACAG
Cloning vector	pET-16b, pASK-IBA3C
Expression vector	pET-16b, pASK-IBA3C
Expression host	*E. coli* BL21 Star
Complete amino-acid sequence of HIS-TEV-*esaAex_2*	HHHHHHSSGENLYFQSGMSQKDSVELDNYINALKQMDSQIDQQSSMQDTGKEEYKQTVKENLDKLREIIQSQESPFSKGMIEDYRKQLTESLQDELANNKDLQDALNSIKMNNAQFAENLEKQLHDDIVKEPDTDTTFIYNMSKQDFIAAGLNEGEANKYEAIVKEAKRYKNEYNLKKPLAEHINLTDYDNQVAQDTSSLINDGVKVQRTETIKSNDINQLTVATDPHFNFEGDIKINGKKYDIKDQSVQLDTSNKEYKVEVNGVAKLKKDAEKDFLKDKTMHLQLLFGQANRQDEPNDKKATSVVDVTLNHNLDGRLSKDALSQQLSALSRFDAHYKMYTDTKGREDKPFDNKRLIDMMVDQVINDMESFKDDKVAVLHQIDSMEENSDKLIDDILNNKKNTTKNKEDISKLIDQLENVKKTFAEEPQEPK

**Table 2:** Crystallization

Method	Hanging drop
Temperature (K)	293
Plate type	Crystalgen
Protein concentration (mg ml^−1^)	5
Buffer composition of protein solution	150 m*M* NaCl, 20 m*M* Tris–HCl pH 8.0
Composition of reservoir solution	0.2 *M* ammonium citrate tribasic pH 7.0, 16% PEG 3350
Volume and ratio of drop	3 µl (1:1 ratio)
Volume of reservoir (µl)	600

**Table 3:** Data collection and processing Values in parentheses are for the outer shell.

Diffraction source	ID30-A3, ESRF
Wavelength (Å)	0.9677
Temperature (K)	100
Detector	EIGER 4M
Crystal-to-detector distance (mm)	308.7
Rotation range per image (°)	0.05
Total rotation range (°)	180
Exposure time per image (s)	0.02
Space group	*P*4_1_2_1_2 or *P*4_3_2_1_2
*a*, *b*, *c* (Å)	197.544, 197.544, 368.334
α, β, γ (°)	90, 90, 90
Mosaicity (°)	0.09
Resolution range (Å)	20.0–4.0
Total No. of reflections	1003682
No. of unique reflections	72074
Completeness (%)	98.6 (97.0)
Multiplicity	13.84
〈*I*/σ(*I*)〉	9.74 (0.98)†
*R*_meas_ (%)	23.9 (276.4)
Overall *B* factor from Wilson plot (Å^2^)	153.1

† CC_1/2_ is 41.6% in the outer shell, indicating that the data contain signal. *I*/σ(*I*) falls below 2.0 at 4.2 Å resolution.
